# A polyvalent coral snake antivenom with broad neutralization capacity

**DOI:** 10.1371/journal.pntd.0007250

**Published:** 2019-03-11

**Authors:** María Carlina Castillo-Beltrán, Juan Pablo Hurtado-Gómez, Vladimir Corredor-Espinel, Francisco Javier Ruiz-Gómez

**Affiliations:** 1 Grupo de Investigación en Animales Ponzoñosos y sus Venenos, Grupo de Producción y Desarrollo Tecnológico, Dirección de Producción, Instituto Nacional de Salud, Bogotá, Colombia; 2 Parasitology Laboratory, Department of Public Health, Faculty of Medicine, Universidad Nacional de Colombia, Bogotá, Colombia; Universidad de Costa Rica, COSTA RICA

## Abstract

Coral snakes of the genus *Micrurus* have a high diversity and wide distribution in the Americas. Despite envenomings by these animals being uncommon, accidents are often severe and may result in death. Producing an antivenom to treat these envenomings has been challenging since coral snakes are difficult to catch, produce small amounts of venom, and the antivenoms produced have shown limited cross neutralization. Here we present data of cross neutralization among monovalent antivenoms raised against *M*. *dumerilii*, *M*. *isozonus*, *M*. *mipartitus* and *M*. *surinamensis* and the development of a new polyvalent coral snake antivenom, resulting from the mix of monovalent antivenoms. Our results, show that this coral snake antivenom has high neutralizing potency and wide taxonomic coverage, constituting a possible alternative for a long sought Pan-American coral snake antivenom.

## Author summary

Coral snakes are distributed in the Americas form Southern United States to Argentina. These snakes cause envenomings that, despite not being common, often lead to death. The antivenoms currently produced to treat accidents caused by these snakes have limitations regarding the number of species venoms they could neutralize. Here, we present an antivenom with a wide spectrum of neutralization, when compared to other Anticoral antivenoms. Nevertheless, more studies are still necessary to evaluate its neutralization capacity against the venoms of other species. This antivenom has great potential, as it neutralizes the lethal effects of some of the most common *Micrurus* species in the Americas.

## Introduction

Coral snakes of the genus *Micrurus* and *Micruroides* represent a highly diverse neotropical monophyletic assembly of about 80 species distributed from the southern United States to northern Argentina [1].

Although uncommon (1–2% of the snake bites in the Americas) [2–4], *Micrurus* envenomation can be lethal due to the presence of potent toxic factors, mainly neurotoxins, causing peripheral paralysis resulting in respiratory failure [5]. The neurotoxic activity of coral snake venoms is mainly due to the presence of non-enzymatic competitive inhibitors of acetylcholine receptors at the neuromuscular junction known as α-neurotoxins of the three-finger (3FTx) protein superfamily and phospholipase A2 (PLA2) enzymes with pre-synaptic activity [5]. These two components have been revealed as the most abundant components in *Micrurus* venoms and vary in their proportion according to the species [5,6].

Snake antivenom production takes several stages and therefore considerable amounts of venom, in order to guarantee the quality of the medicament [7–10]. First, the toxicity of the venoms used for immunization must be determined (e.g. median lethal dose), then, animals (i.e. horses, goats) are inoculated with non-lethal doses of venom to produce a hyperimmune serum and subsequently, potency trials (e.g. median effective dose) must be carried out at different times in order to test the efficacy and stability of the product [9,10]. *Micrurus* snakes have relatively small sizes, which results in low venom yields, are difficult to find in the field and to maintain in captivity for extended periods of time. These aspects constitute serious setbacks for gathering sufficient amounts of venom for the production of coral snake antivenoms [11].

Antivenoms capable of neutralizing the toxic activities of a large range of heterologous *Micrurus* venoms have been long sought. Initially, as a mean to use antivenoms derived from snakes capable of yielding large amounts of venom against the toxic activities of snakes considered a public health threat but yielding very low amounts of venom per individual [12,13]. Later, as a way to produce antivenoms capable of neutralizing the lethal activities of a wide range of coral snake venoms that could be used in the Americas [8]. However, although antibody cross-reactivity has been widely observed between monovalent antisera and heterologous *Micrurus* venoms, in many cases resulting the ability of the antivenom to neutralize the lethal activity of the heterologous venom [14–16], in a number of cases and despite cross-reactivity, antivenoms are unable to neutralize the lethal effect of heterologous venoms [8,15,17,18]. In the Americas, anti-coral snake antivenoms are produced by the Instituto Nacional de Producción de Biolo’gicos (ANLIS) “Dr Carlos Malbrán” in Argentina, the Clodomiro Picado Institute (ICP) in Costa Rica, the Butantan Institute in Brazil, Instituto Bioclon in Mexico [19] and Laboratorios Probiol in Colombia [20]. However, while the antivenoms produced in Central America can neutralize the lethal activities of *M*. *nigrocinctus*, *M*. *mosquitensis*, *M*. *dumerilii*, *M*. *fulvius*, *M*. *clarki*, *M*. *alleni* and *M*. *tener*, they are unable to neutralize the lethal activities of *M*. *mipartitus*, *M*. *surinamensis*, *M*. *spixii and M*. *pyrrhocryptus* [21–24]. Likewise, those produced in South America, while able to neutralize the lethal activities of *M*. *frontalis*, *M*. *corallinus*, *M*. *pyrrhocryptus*, *M*. *fulvius*, *M*. *nigrocinctus* and *M*. *surinamensis*, are unable to neutralize the lethal activities of *M*. *altirostris*, *M*. *ibiboboca*, *M*. *lemniscatus* and *M*. *spixii* [25–27].

Based on the large extent of cross-reactivity between elapidic antivenoms and elapidic heterologous venoms and the cross neutralization of the lethal activity of a *Notechis scutatus* antivenom against the lethal activity of the *M*.*fulvius* venom [28], polyvalent anti-elapidic antivenoms have thus been considered as an alternative for the long sought development of a Pan-American anti-coral snake antivenom. In fact, a pentavalent anti-elapidic antivenom developed by CSL Limited in Australia using as antigens *Notechis scutatus*, *Pseudechis australis*, *Pseudonaja textilis*, *Acanthophis antarcticus* and *Oxyuranus scutelatus* venoms has been shown to neutralize the lethal activities of *M*. *corallinus*, *M*. *frontalis*, *M*. *fulvius*, *M*. *nigrocinctus* and *M*. *pyrrhocryptus* [29].

Here we report the production of a horse polyvalent anti-coral (*Micrurus*) snake antivenom derived from the mixing of monovalent antivenoms against *M*. *dumerilii*, *M*. *mipartitus*, *M*. *isozonus* and *M*. *surinamensis* venoms. The polyvalent antivenom is capable of neutralizing the lethal activity of *M*. *dumerilii*, *M*. *mipartitus*, *M*. *isozonus*, *M*. *surinamensis*, *M*. *medemi*, *M*. *lemniscatus* and *M*. *spixii* venoms thus constituting a promising Pan-American anti-coral antivenom.

## Materials and methods

### Venom source and choice

**Fig 1:**
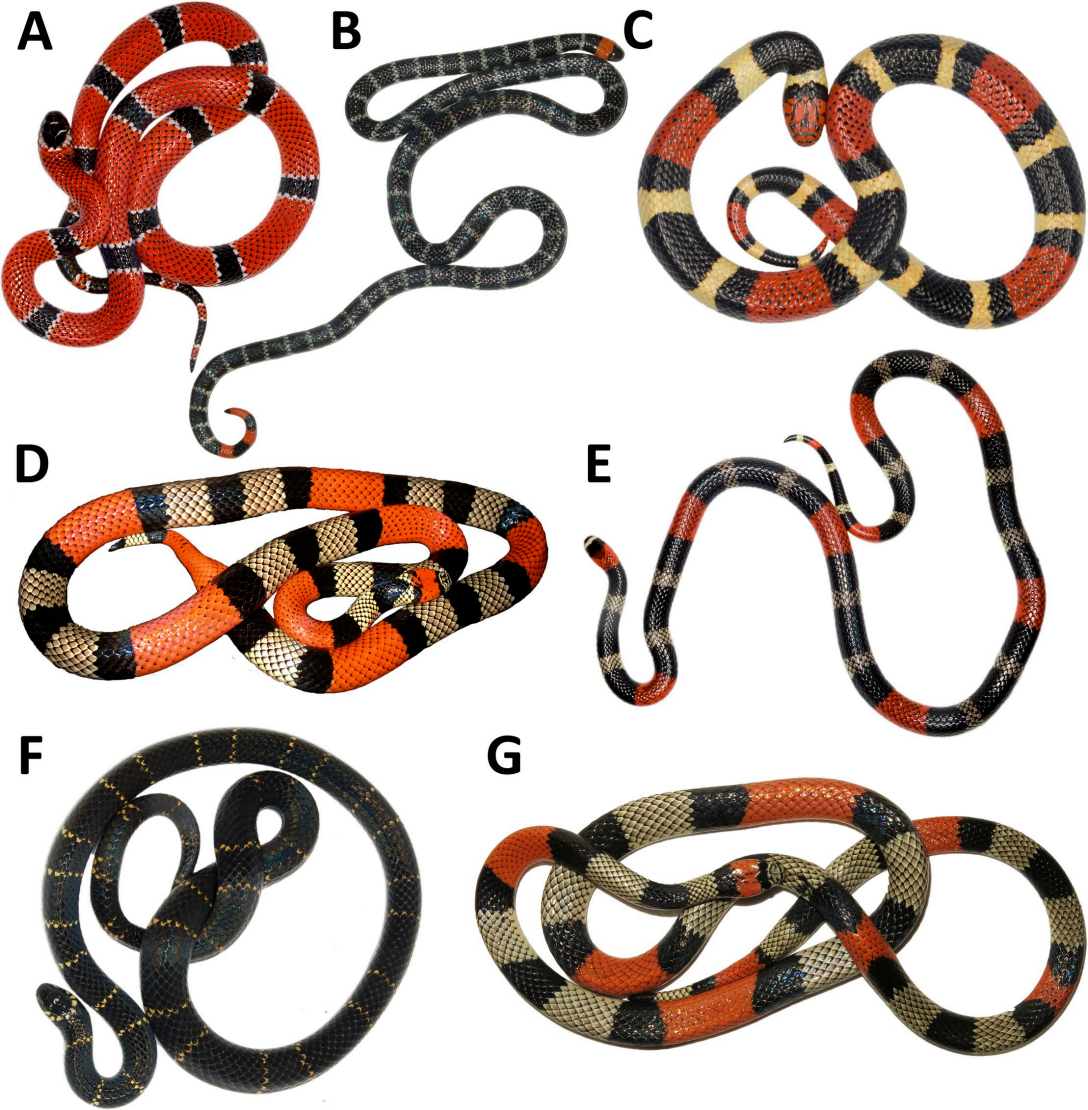
Photographs of *Micrurus* species studied herein. A. *M*. *dumerilii*; B. *M*. *mipartitus*; C. *M*. *surinamensis*; D. *M*. *isozonus*; E. *M*. *lemniscatus*; F. *M*. *medemi*; G. *M*. *spixii*. Photographs: A-C, E-G, JPHG; D, Jairo Maldonado-García.

The lyophilized venoms were obtained from the venom bank at the Instituto Nacional de Salud (INS) de Colombia, Bogotá. Venoms were kept frozen at -40°C. Species included in the study were chosen based on venom availability and inclusion on different *Micrurus* phyletic lineages [30,31]: *M*. *mipartitus* (Middle Magdalena Valley–MMV) of the bicolored group; *M*. *dumerilii* (MMV), *M*. *medemi* (Orinoco Basin—OB) from the monadal group and *M*. *isozonus* (OB), *M*. *lemniscatus* (OB), *M*. *surinamensis* (OB) of the triadal group (Fig 1). All venoms used were obtained from Colombian specimens.

### Animals

Eight mixed breed horses were used with weights between 325–370 kg and between four to six years old. Horses were kept in the open, in pasture enclosures in a farm of the INS in Bojacá, Cundinamarca, Colombia, under veterinary care. Horses were vaccinated against tetanus and equine influenza, dewormed for gut helminths and washed to remove potential external parasites. Hematological, hepatic and kidney health was tested every six months and only horses with healthy organs until the last inspection were used for immunization. Mice CD-1 ICR strain, of 16–20 g, were obtained from the animal facility at the INS, Bogotá.

### Ethics statement

Experiments followed ethical procedures established in the protocols for animal experimentation at the INS (INT-R04.0000.01) and by the World Health Organization [9,10]. Animal experimentation was approved by the Institutional Committee for Animal Use and Care at the National Health Institute (Comité Institucional para el Cuidado y Uso de los Animales en el Instituto Nacional de Salud -CICUAL-INS), resolution 0052 of 2018.

### Antivenom production

Hyperimmune horse sera was obtained following the World Health Organization (WHO) guidelines [9,10] and the internal immunization protocol defined by the INS. In order to evaluate the immunogenicity of individual venoms and the capacity of individual antisera to cross neutralize heterologous venoms, experimental monospecific antivenoms were produced with the venom of four *Micrurus* species: *M*. *dumerilii*, *M*. *isozonus*, *M*. *mipartitus* and *M*. *surinamensis*. For each species venom, two horses were used. The immunization scheme for each horse lasted for up to three months, with injections administered every 5 to 15 days. For the first immunization, the venom was dissolved in Freund’s adjuvant (Becton Dickinson), whereas the remaining ones were dissolved in saline solution 0.85% (SS). Each injection had a volume between 0.5–5 mL, with 15–20 mg of venom, depending on the venom´s toxicity. Once the immunization scheme was completed, animals were bled to test whether there were quantifiable titers following neutralization procedures (see below). When appropriate antivenom neutralization titers were attained (≥3 LD_50_), horses were bled through puncture in the jugular vein. Up to eight liters of blood were collected in sterile plastic bags with anticoagulant, and plasma separation from cells was made by gravity. Cells were subsequently reinjected back into the horses for a better and faster recovery. Plasma was subsequently purified by means of precipitation with ammonium sulfate and sterilizing filtration, in order to obtain the concentrated antivenom immunoglobulin solution and stored at 2–8°C [32].

Polyvalent antivenom was produced by mixing of monovalent antivenoms and diluted to reach neutralization titers of 0.3 mg/mL of *M*. *dumerilii* and *M*. *surinamensis*, 0.8 mg/mL of *M*. *mipartitus* and 2 mg/mL of *M*. *isozonus*. This antivenom corresponds to the "Antiveneno Anticoral Polivalente", produced by the Instituto Nacional de Salud (INS), batch number 15AMP01, with expiration date of March of 2018.

### Determination of protein content

Protein concentration was determined by the Kjeldahl method [10,33], following standardized protocol INS (MEN-R04.6020–010). Values correspond to grams per 100 mL and are expressed as percentage.

### Biological activities

#### Lethality

Venom lethality was estimated using the median lethal dose (LD_50_), following WHO guidelines [9] and INS standard internal protocols. Serial dilutions of venom dissolved in 500μL of SS were injected intraperitoneally in mice (n = 5 per dose). Seven to eight dilutions were tested for each venom, with dilution factors ranging from 1.5 to 1.7 with concentrations ranging from 1.85 to 85.43μg/mice. A negative control consisting of 500 μL of SS was used in each trial. Death ratio was read after 48 hours and the experiments were considered valid only when reaching values of both, zero and 100%. The LD_50_ and respective 95% confidence intervals were established using the Spearman-Kärber method [34,35] and was expressed in micrograms of venom (μg) per mice. For comparison purposes, LD_50_ values from literature were transformed to μg/mice, using the mean of the weight range of the mice used.

#### Neutralization

Neutralization capability of monovalent and polyvalent antivenoms was determined using the median effective dose (ED_50_), following WHO guidelines [9,10,36] and INS standard internal protocols. Solutions containing different concentrations of each monovalent or polyvalent antivenoms were mixed with 3LD_50_/mice of venom from each species (as obtained in the lethality assays), preincubated at 37°C for 30 minutes and then injected intraperitoneally in mice (n = 5 per dose, 500 μL/mice). Five to six different dilutions of the antivenoms were tested, with dilution factors ranging between 2.6 to 3.3 and attaining concentrations of 0.08 to 32.93 mg/mL. Three control groups were used, two negative (one with antivenom and one with saline solution, 500 μL/mice) and one positive (3 LD_50_ of venom/mice). Death ratio was read after 48 hours and experiments were only considered valid when attaining death ratios of both, zero and 100%. The ED_50_ was established using the Spearman-Kärber method [34,35] and expressed in milligrams (mg) of venom per milliliter (mL) of monovalent or polyvalent antivenom. Neutralization, was also expressed as the number of LD_50_ per 1 mL of monovalent or 10 mL of polyvalent antivenom (the volume of a commercial vial of antivenom). For comparison purposes, ED_50_ values from the literature were transformed to the number of LD_50_ per 10mL of antivenom, using the LD_50_ values here obtained or cited in the corresponding study.

We classified the neutralization capability of the monovalent fractions and the polyvalent antivenom according the number of LD_50_ values neutralized, as follows:

- Low: Less than three LD_50_ (#LD_50_ < 3) (the value equivalent to the number of LD_50_ used as challenge in the neutralization assay, which is the minimum neutralization titer expected for an antivenom).
- Moderate: Between three and less than 60 LD_50_ (3 ≤ #LD_50_ < 60) (the number corresponding to the minimum number of LD_50_ used for challenge and the minimum number of LD_50_ neutralized by a monovalent antivenom against its homologous venom).
- High: 60 or more LD_50_ (#LD50 ≥ 60) (the neutralization capability above the minimum number of LD_50_ neutralized by a monovalent antivenom against its homologous venom).

Because monovalent fractions are more concentrated than the antivenom, values for monovalent fractions were considered for 1 mL, whereas those for the polyvalent antivenom were considered for 10 mL.

## Results

### Protein content

Protein content for antivenoms were 10.8% for anti-*dumerilii*, 8.2% for anti-*mipartitus*, 9.3% for anti-*isozonus*, 9.4% for anti-*surinamensis* and 8.1% for the polyvalent.

### Lethality

Venoms derived from the seven species studied showed a wide variation in lethality. Venom from *M*. *mipartitus* showed the lowest lethality (1.87 μg/g), whereas *M*. *isozonus* (0.35 μg/g) venom displayed the highest one (Table 1).

**Table 1:** LD_50_ values from *Micrurus* venoms studied herein, compared with literature data.

	*Micrurus s*pecies						
Source	*dumerilii*	*mipartitus*	*isozonus*	*surinamensis*	*medemi*	*lemniscatus*	*spixii*
This study	23.72	33.62	6.29	29.17	8.79	22.87	13.89
	(18.09–31.12)	(26.15–43.22)	(5.14–7.69)	(24.42–34.84)	(7.16–10.80)	(17.79–29.4)	(N.D.)
Bolaños et al. [37]	17.00	-	-	5−10	-	5*	10−15*
	(N.S.)			(N.S.)		(N.S.)	(N.S.)
Tanaka et al.[16]		-	-	58**	-	13**	8**
				(43–87)		(7–22)	(6–16)
Silva[38]		-	-	9.43	-	10.12	50.6
				(N.S.)		(N.S.)	(N.S.)
Cohen [14]	16.3	-	-	-	-	-	27.3***
	(N.S.)						(N.S.)
Cohen[13]	11.9	-	-	-	-	-	-
	(N.S.)						
Rey-Suarez et al. [23]	22.42	-	-	-	-	-	-
	(14.5–36–1)						
Otero et al. [39]	-	9	-	-	-	-	-
		(6.6–9.1)					
Oliveira et al. [40]	-	-	-	14**	-	-	-
				(11.3–16.8)			
de Roodt et al. [26]	-	-	-	7.6	-	-	-
				(5–19)			
de Roodt et al. [41]	-	-	-	20	-	-	-
				(N.S.)			
Salazar et al. [42]	-	-	11.4	-	-	-	-
			(N.S.)				
Higashi et al. [15]	-	-	-	-	-	-	6.7**
							(N.S.)
Max/Min ratio	1.99	3.74	1.81	11.60	-	4.57	7.55

Values are given in μg/mice (see Materials and Methods for details). 95% confidence intervals are indicated in parenthesis. N.D., not determined because, on the trial doses, only death ratios corresponding to 0% and 100% were recorded; N.S., not specified on the study. Data without asterisk marks correspond to venoms from Colombia;

*unknown origin;

** Brazil;

*** Peru.

### Neutralization by monovalent antivenoms

Monovalent antivenoms showed appropriate neutralization titers against homologous venoms. *M*. *dumerilii* and *M*. *isozonus* showed the lowest and highest titers, respectively (Table 2). The anti-*dumerilii* antivenom neutralized the lethality of *M*. *isozonus* and *M*. *mipartitus* venoms, with higher titers than those against the homologous venom, but with low titers against *M*. *surinamensis* venom. Anti-*mipartitus* antivenom showed low neutralization activity against *M*. *dumerilii* and moderate against *M*. *isozonus* and *M*. *surinamensis* venoms. The anti-*isozonus* antivenom displayed low neutralization titers against *M*. *dumerilii*, moderated against *M*. *surinamensis* and high against *M*. *mipartitus* venoms. Finally, the anti-*surinamensis* antivenom showed low neutralization capability against all heterologous venoms.

**Table 2:** Neutralization efficacy of monovalent and polyvalent antivenoms.

	Neutralization measurement	*Micrurus* species venom						
		*dumerilii*	*mipartitus*	*isozonus*	*surinamensis*	*medemi*	*lemniscatus*	*spixii*
Anti- *dumerilii*	ED_50_	1.86	2.14	3.17	<0.11	-	-	-
		(0.9–3.84)	(0.96–4.75)	(1.12–5.24)				
	#LD50/mL	78.42	63.65	503.97	<3.60	-	-	-
Anti- *mipartitus*	ED_50_	<0.09	>2.36	0.3	0.17	-	-	-
				(0.13–0.7)	(N.D.)			
	#LD50/mL	<3.58	>70.20	47.69	5.83	-	-	-
Anti- *isozonus*	ED_50_	<0.09	>2.36	>7.89	0.17	-	-	-
					(N.D.)			
	#LD50/mL	<3.58	>70.20	>1254.37	5.83	-	-	-
Anti- *surinamensis*	ED_50_	<0.09	<0.12	<0.06	1.87	-	-	-
					(0.92–3.8)			
	#LD50/mL	<3.58	<3,57	<8.74	64.11	-	-	-
Polyvalent Antivenom	ED_50_	0.36	0.94	2.24	0.31	0.68	0.58	1.58
		(N.D.)	(0.68–1.28)	(1.47–3.4)	(0.2–0.48)	(0.44–1.06)	(0.4–0.84)	(N.D.)
	#LD50/mL	15.18	27.96	356.12	10.63	77.36	25.36	113.75
	mg AV protein/1 mg venom	225.00	86.17	36.16	261.29	119.12	139.66	51.27

Results are expressed both as mg/mL (ED_50_) and number of LD_50_ neutralized per mL of antivenom (#LD_50_/mL) and for the polyvalent antivenom the mg of antivenom protein necessary to neutralize 1 mg of venom. 95% confidence intervals are indicated in parenthesis. N.D., not determined because, on the trial doses, only death ratios corresponding to 0% and 100% were recorded.

Neutralization by the polyvalent antivenom

The antivenom showed a high capacity of neutralizing the effect of both homologous and heterologous venoms (Table 2). Neutralization capacity against homologous venoms, was lowest against *M*. *surinamensis*, and highest against *M*. *isozonus*. The antivenom was able to neutralize the lethal effects of heterologous venoms derived from *M*. *spixii* (1.58 mg/mL), *M*. *lemniscatus* (0.58 mg/mL) and *M*. *medemi* (0.68 mg/mL). Surprisingly, its neutralization titers against the heterologous venoms tested were higher than the titers against the homologous venoms derived from *M*. *dumerilii* and *M*. *surinamensis*. Noteworthy, the neutralization titer against the *M*. *spixii* venom was the second highest (Table 2).

## Discussion

### Protein content

Protein content of some of the monovalent antivenoms surpass the upper limit of 10% recommended by WHO [10](e.g. anti-dumerilii 10.8%). Nevertheless, the polyvalent antivenom used as therapy, has a protein content below this limit (8.1%). This value is higher than the 5.5% reported for the antivenom produced by the Instituto Nacional de Producción de Biológicos, Argentina and 4% reported for the Coralmyn, Bioclon, Mexico[26]. Such differences in protein content might be associated to the polyvalence of the antivenom and to the relatively high neutralization titers. It is believed that high protein concentration might increase the probability of adverse reactions [10]. Additionally, the relatively high neutralization titers compensate for this, since less medicament is required, therefore diminishing the total amount of protein administered to the patient.

### Lethality

Our results show a wide variation within the seven venoms tested and important differences as compared with the LD_50_ values found for the same species in other studies (Table 1). Estimations of the LD_50_ for the venom of a given species varied within studies, to the extent that the maximum value was almost 12 times the value of the minimum measurement (i.e. *M*. *surinamensis*; Table 1). It is difficult to explain the amount of variability within a species, given the number of variables that may influence the final results. Methods to estimate LD_50_ values vary according to several factors: mice weight and strain, volume of administration, venom treatment (e.g. dried vs lyophilized), inoculation route (e.g. intravenous vs intraperitoneal), etc. All these variables have proven to influence the final results [43]. On the other hand, differences in venom lethality may be the result of geographical variation [5]. For example, the venoms of *M*. *dumerilii* in this and other studies come from the middle Magdalena River Valley region of Colombia and LD_50_ are relatively similar among studies (Table 1). In the case of *M*. *surinamensis*, where LD_50_ varied widely, venoms originated from specimens captured over a large geographical distribution in the Orinoco and Amazonas basins [26,37,38]. Different regions may differ in many aspects (e.g. climate, geography) that may influence venom quality. Moreover, results by the same authors [26,41] for *M*. *surinamensis* from the same region, apparently using the same methodology, reached different results (Table 1). Therefore, at this point, conclusions regarding what is influencing differences in venom lethality may be hasty. Future efforts should be made to standardize procedures among laboratories in order to get comparable results.

### Neutralization by monovalent antivenoms

As shown here, previous works found that monovalent antivenoms neutralize the lethal effects of homologous venoms [8,13,14,44] (Table 2). All monovalent antivenoms described in this study showed some degree of cross neutralization. Likewise, Cohen and collaborators [13,14], produced experimental monovalent antivenoms in rabbits by immunization with the venom of *M*. *dumerilii*, reaching high titers when neutralizing the homologous venom and moderate titers against two (*M*.*fulvius* and *M*. *spixii*) out of the seven venoms studied. In our trials, all monovalent antivenoms showed low neutralization titers against the lethal effect of *M*. *dumerilii* venom, contrary to other reports showing that this venom was neutralized by three (*M*. *frontalis*, *M*. *fulvius*, *M*. *nigrocinctus*) out of the four heterologous monovalent antivenoms tested [13,14]. On the other hand, the anti-*surinamensis* serum, as reported by several studies, showed low cross-neutralization [44]. Herein, we tested for the first time the neutralization capability of *M*. *mipartitus* and *M*. *isozonus* monovalent antivenoms: the first only showed high cross neutralization titers against *M*. *isozonus* and the second only against *M*. *mipartitus* (Table 2). It should be noted that Cohen and collaborators [14] tested the anti-*dumerilii* antivenom against the venom of a subspecies called *M*. *mipartitus hertwigii*, but this taxon is currently recognized as *M*. *multifasciatus* [45].

Our results show that cross neutralization does not operate in both directions. As stated before, anti-*dumerilii* antivenom showed high titers against *M*. *mipartitus* and *M*. *isozonus*, but low titers were recovered from anti-*isozonus* antivenom against *M*. *dumerilii* venom (Table 2). This observation is not new, other works using monovalent antivenom have found similar results [8,13,14,44]. This is an important fact that must be accounted for when designing antivenoms or eventually, when choosing antivenoms for envenomation treatments. For example, the antivenom produced in Costa Rica, which is produced using *M*. *nigrocinctus* venom as an antigen, neutralizes the lethality of *M*. *dumerilii* [23], one of the coral snakes involved in a large proportion of coral snake bite accidents in Colombia but the anti-*dumerilii* monovalent antivenom does not neutralize the activities of the *M*. *nigrocintus* venom [14].

### Neutralization by the polyvalent antivenom

Our data shows that the INS coral antivenom has good direct and cross neutralization titers (Tables 2 and 3). Particularly, the neutralization titers against all the heterologous venoms were higher than those against the homologous *M*. *dumerilii* and *M*. *surinamensis* venoms, as measured by either the amount of venom or the number of neutralized LD_50_s. Currently available Latin American coral snake antivenoms have shown different neutralization capabilities. The Brazilian, Instituto Butantan (raised against *M*. *corallinus* and *M*. *frontalis*), has proven to properly neutralize the venom of five species, but was ineffective against five [16,29,44,46]. Costa Rican monovalent antivenom (anti*M*. *nigrocinctus*), produced by Instituto Clodomiro Picado, has shown to be efficient against five species but unable to neutralize the venom of other two [21–24,47,48]. Mexican Coralmyn monovalent antivenom (against *M*. *nigrocinctus*), manufactured by Bioclon Laboratory, neutralizes the venom of three species, but is ineffective against four [25–27]. Finally, the monovalent Argentinian antivenom (raised against *M*. *pyrrhocryptus*) produced by Instituto Nacional de Productos Biológicos, has been shown to neutralize the venom of four species, but unable to neutralize the venom of other two [26]. The INS antivenom presented herein has wide neutralization capability against seven species. Further neutralization experiments against a wide range of *Micrurus* venoms are highly desirable.

**Table 3:** Comparative ED_50_^a^ values of the coral snake antivenoms distributed in South America, against venoms of the species studied herein.

		*Micrurus* species venom						
Producer	Source^b^^,^ ^c^	*dumerilii*	*mipartitus*	*isozonus*	*surinamensis*	*medemi*	*lemniscatus*	*spixii*
**INS**	This study	3.6	9.4	22.4	3.1	**6.8**	**5.8**	**15.8**
		(151.77)	(279.6)	(3561.21)	(106.27)	**(773.61)**	**(253.61)**	**(1137.51)**
**ICP**	Rey Suarez *et al*. [23,47]	**2**	**none**	-	-	-	-	-
		**(89.21)**						
**Bioclon**	de Roodt *et al*. [26]	**-**	**-**	-	**<0.3**	**-**	-	-
					**(<39.47)**			
**IB**	Ramos *et al*. [29]	**-**	**-**	-	-	**-**	**none**^**d**^	**none**^**d**^
	Tanaka *et al*. [16]	**-**	**-**	-	-	**-**	**0.7**	**5**
							**(53.85)**^**e**^	**(625)**^**e**^
	Tanaka *et al*. [44]	**-**	**-**	-	-	**-**	**0.7**	**3.7**
							**(53.85)** ^**e**^	**(462.50)** ^**e**^
**INPBA**	de Roodt *et al*. [26]	**-**	**-**	-	**4.22**	**-**	-	-
					**(555.26)**			

^a^ ED_50_ is given as mg/10 mL vial (#LD50 [μg/mice]/ 10 mL vial).

^b^ All studies challenged antivenoms against 3 LD_50_ except Tanaka *et al*. [16,44] who used 2 LD_50_.

^c^ In all studies mixtures of venom and antivenom were injected intraperitoneally.

^d^ No ED_50_ was estimated, only ratio of survival from one trial.

^e^ Values are estimates from figures since authors do not provide exact values.

INS, Instituto Nacional de Salud, Colombia; ICP, Instituto Clodomiro Picado, Costa Rica; IB, Instituto Butantan, Brazil; Bioclon, Laboratorios Biolclon, Mexico; INPBA, Instituto Nacional de Producción de Biológicos, Argentina. Values in bold indicate heterologous venoms for the given antivenom. See Material and Methods for additional details.

The different neutralization range between the INS antivenom and other Latin American antivenoms is likely associated to the fact that most *Micrurus* antivenoms are mono or bivalent, whereas the INS is a mixture of antibodies raised against four phylogenetically different species. An early experimental polyvalent antivenom produced by Bolaños *et al*. [37] showed somehow similar results. This antivenom was raised against venoms derived from *M*. *pyrrhocryptus* (referred as *M*. *frontalis pyrrhocryptus*), *M*. *multifasciatus* (referred as *M*. *mipartitus hertwigi*) and *M*. *nigrocinctus*; and was able to neutralize the lethal effect of homologous and heterologous venoms (*M*. *fulvius*, *M*. *dumerilii*, *M*. *frontalis*, *M*. *corallinus*, *M*. *spixii*, *M*. *mipartitus*, *M*. *alleni* and *M*. *lemniscatus*. However, it was unable to neutralize the venom from *M*. *surinamensis*. Contrarily, an experimental polyvalent antivenom produced by Tanaka *et al*. [44], as a mixture of monovalent antivenoms raised against *M*. *spixii*, *M*. *frontalis*, *M*. *corallinus*, *M*. *altirostris* and *M*. *lemniscatus*, showed limited neutralizing efficacy.

Antivenoms from Brazil, Costa Rica and Mexico have not included the venom of *M*. *surinamensis* in their immunization schemes, and have very low or no neutralization capacity against this venom. The antivenom we developed includes the venom of this species in the immunization scheme, and displays high neutralization titers against the lethal effects of the *M*. *surinamensis* venom (Table 3). Given the particularities of this venom and the inability of heterologous antivenoms to neutralize *M*. *surinamensis* venom, the inclusion of venom derived from this species as an immunogen is important in the production of antivenoms in countries where this species occur, such as Brazil, Ecuador, Peru and Venezuela, in order to provide proper therapeutic alternatives [49]. Surprisingly, the commercial monovalent antivenom produced in Argentina, raised against *M*. *pyrrhocryptus*, proved to be effective against this species, which is another therapeutic alternative for this difficult to neutralize species venom (Table 3) [26]. Another antivenom that apparently neutralizes the venom of *M*. *surinamensis* is the one produced by Probiol [20]. This antivenom, derived from the immunization with *M*. *lemniscatus*, *M*. *spixii* and *M*. *surinamensis* venoms, claims to neutralize the venoms from *M*. *mipartitus*, *M*. *surinamensis*, *M*. *dumerilii*, *M*. *medemi* and *M*. *spixii* [20]. Nevertheless, the titers of neutralization are not known and no information is provided for the neutralization capacity against the homologous venom from *M*. *lemniscatus*.

When comparing the INS antivenom neutralization capacity against the species tested with other antivenoms, INS antivenom showed higher titers with respect to both the amount of venom and the number of median lethal doses neutralized, except for *M*. *surinamensis* which is more efficiently neutralized by the antivenom from the Instituto Nacional de Producción de Biológicos, Argentina. (Table 3). All studies here compared appraised the neutralization ability of antivenoms against three LD_50_, except for Tanaka et al. [16,44], that challenged against two, which might imply that Butantan’s antivenom might have lower neutralization capability. Additionally, our results proved that the INS antivenom neutralizes with high efficacy the lethality of a broad range of *Micrurus* species venoms (Table 3). These properties are desirable in the clinical practice. First, because with such titers, less amounts (volume and protein) of medicament are needed and the probability of adverse reactions reduces. Second, because a wide taxonomic coverage is always desired, since most of the time there is no appropriate identification of the species causing the accidents.

Comparisons among the neutralization capabilities of antivenoms, as for LD_50_ toxicity measurements, is difficult. Trials among studies vary widely in methodological aspects like the strain of mice, weight, challenging doses (i.e. from 2–5 LD_50_), value determination method (e.g. Spearman-Kärber, Probits) or route of injection (e.g. intraperitoneal vs. intravenous). Nevertheless, even if neutralization values vary, the fact that the tested antivenoms are or are not able to neutralize the studied venoms is hardly obscured.

The outcomes of this study show that INS antivenom is the best therapeutic alternative to treat coral snake envenomation in Colombia. Furthermore, this antivenom is the closest version of a long sought Pan-American anti-coral snake antivenom. Because most of the coral snake species whose venoms are neutralized by this antivenom are present in other south American countries, where no coral snake antivenom is produced, like Ecuador, Peru and Venezuela [50,51], or even Brazil, where the antivenom produced has a restricted efficacy for some species [16,44], this antivenom represent a treatment alternative for coral snake envenomation. Additionally, this antivenom might work in North America, given that cross neutralization of anti-*M*. *dumerilii* antivenom against *M*. *fulvius* venom has been reported [13,14]. On the other hand, the ability to neutralize the venom of Central American species remains to be proven, since only anti-*dumerilii* antivenom have been tested against *M*. *nigrocinctus* venom with negative results [14].

As aforementioned, the design of coral snake antivenoms has been hampered by low venom yields and unpredictable cross neutralization. Production of monovalent experimental antivenoms, evaluation of cross neutralization capacity and finally mixing of appropriate monovalent antivenoms to the desired neutralization titers is an effective approach for the production of polyvalent antivenoms. This way, producers might maximize limited resources (venom) while gaining knowledge on venom immunogenicity and sera cross reactivity.

Despite our promising results, various aspects must be accounted for. Around eighty species of *Micrurus* occur in the Americas, of which close to 30 occur in Colombia. We have tested the neutralization capacity against the venoms of only seven species. Even if those are the ones more often involved in accidents, there is a substantial number of questions that require our understanding. Examples of this are the spectrum of neutralization of this antivenom, the neutralization capacity against independent activities, such as neurotoxicity and myotoxicity and the best formulation of venom combinations required to produce an antivenom with high and broad neutralization capacities. An understanding of these aspects might also come from clinical results. Finally, this warrant large collaborative efforts to standardize neutralizations tests for comparative purposes and test anti-coral snake antivenoms produced in the Americas against a large number of *Micrurus* venoms.
